# Concluding Remarks

**DOI:** 10.4103/0973-1229.32157

**Published:** 2007

1. Private investment in biomedical research has increased over the last few decades. At most places it has been welcomed as the next best thing to technology itself. There are significant costs involved too. Major areas of conflict of interest especially applicable to biomedical research have arisen, as academia interacts with industry.
2. Pharma image stinks because of a number of errors of omission and commission. A recent example is suppression of negative findings about Bayer's Trasylol (Aprotinin) and the marketing maneuvers of Eli Lilly's Xigris (rhAPC).
3. A voluntary moratorium over pharma spending to pamper drug prescribers is necessary.
4. The integrity of industry-sponsored clinical research has come under increasing scrutiny. The basic shizm is between the value system of a patient welfare driven professional and that of a profit driven industry. While one wants to avoid control but wants the dough, the other wants to exercise the control by supplying the dough.
5. Clinical practice guidelines (CPG) are considered important as they guide diagnostic/therapeutic regimen of a large number of medical professionals and hospitals and provide recommendations on drugs, dosages and criteria for selection. Besides clinical trials, they are another area of growing influence by the pharmaceutical industry. For example, in a recent survey it was found that about 60% of 192 authors of clinical practice guidelines reported they had financial connections with the companies whose drugs were under consideration. This finding casts serious doubt on the credibility of this important pillar of modern clinical practice. It needs urgent reparative action. One of them is prospective and retrospective disclosure of financial conflict of interest by authors of CPGs.
6. A Conference on Guideline Standardization (COGS) was convened in April 2002 ′to define a standard for guideline reporting that would promote guideline quality and facilitate implementation’. It includes items for standardization, conceptual issues, up gradation, conflict of interest, patient interest and systematization. Even items for individual preferences, choice or values are not neglected. Special mention must be made of items that specify disclosure of conflict of interest both in the Developer (including the organization that develops and the individuals involved in the guideline's formulation), as well as in the sponsor or funding source (and its role in developing and/or reporting the guideline).
7. Recommendations of CPGs and CDR panels are conflicting. One considers effectiveness, the other cost-effectiveness. However, CPGs do not adhere to established methodological standards; critical information that would attest to validity is regularly absent; explicit criteria to grade scientific evidence that supports their recommendations is absent from 82% of guidelines; 87% are not in a position to report whether a systematic literature search was performed; 67% do not describe the type of professionals used in guidelines development; and there is marked variation in the quality of guidelines. Moreover, CPG guideline layers often are conflicted in their interests. The problem can be resolved to a large extent by taking a simple step: making CPG panelists go into cost effectiveness along with recommending Guidelines. What then happens is they have to consider not only effectiveness but also costs. Effectiveness can be fudged, cost cannot. Why? Because, what is the cost is well known. Therapies in Guidelines should be recommended and graded according to whether they are Most, Moderately or Least Cost Effective. For that CPGs will have to perform an economic analysis as well. This will meet with resistance for obvious reasons.
8. Since guideline groups cannot be kept on the straight and narrow path and till we find fool proof ways of keeping them thus, we have no option but to stress that under no circumstances they can mislead, or get misled themselves, in the name of patient advocacy, to recommend costly new therapies which have still not proved their effectiveness conclusively, nor get away with conflicted recommendations, which it is still not obligatory to reveal. A simple rider like making it mandatory for guideline groups to go into both effectiveness and cost effectiveness takes care that this is ensured.
9. The AGREE Collaboration (AGREE stands for Appraisal of Guidelines, Research and Evaluation) has created and validated tools by which clinicians can themselves rate guidelines by identifying factors that determine their quality. To facilitate this process, a body like the GAC (Guidelines Advisory Committee) applies the AGREE criteria to individual guidelines and rates and endorses the best possible guideline.
10. Clinical Practice Guidelines are another example of an excellent idea likely to go to seed due to sponsor manipulation and forces of the market place camouflaging as evidence based medicine. The need to weed out conflicted experts and make the process of therapy selection transparent must go hand in hand with laying down clear-cut criteria for guideline formulation and rejection of conflicted submissions by vigilant journal publication policies and editors.
11. A number of disease-specific foundations are heavily funded by industry that creates serious conflict of interest likely to result in favourably recommending therapies of sponsors. The case of Alteplase and the American Heart Foundation is a recent example.
12. Whistle blowing papers and research to expose misdemeanours must be undertaken and journals and their editors should welcome such papers as legitimate research, without encouraging a witch-hunt.
13. The USP of journals and researchers is credibility. Credibility can be marketed, but it can't be bought. Sponsors will be forced to seek such credibility without allowing them to compromise it. Such is the game journal publishers/editors and genuine researchers will have to play with sponsors. But they can do so only if they are thoroughly competent and have abiding faith in an uncompromising set of ethical values.
14. The Task Force of the AAMC, in its two reports of 2001 and 2002, recognises the necessity and inevitability of the academia-industry connect, but wants to avoid its undesirable influence on the integrity of research and the welfare of human research subjects. It is specially concerned that public, activist and governmental control and concern does not cast a spanner in the works of a potentially promising relationship.
15. Constant pampering by sponsors has dulled most drug prescribers’ critical capabilities. Sponsorship is a potent anaeasthetic to many ethical concerns. It can blunt the thrust of many a sabre-rattling critic.
16. Both medical associations and research journal editors are getting concerned with individual and institutional conflicts of interest in the conduct of clinical research and documents are now available which address these issues.
17. Edits are concerned with whether academic medicine is for sale (Angell, in *N Engl J Med* 2000); what are the controlling interests of research (Editorial, *CMAJ,* 2002a); how the invisible hand of the marketing department works (Editorial, *CMAJ*, 2002b); how contracts affect institutions and academic freedom (Drazen in *N Engl J Med*, 2002); what are the choices for the academic medical center in collaborating with industry - (Moses et al in *N Engl J Med*, 2002); how does one ensure integrity of scientific research (Editorial, *Lancet*, 2002); how one maintains public trust in clinical research (Kelch in *N Engl J Med*, 2002); how does one ensure academic freedom in clinical research (Nathan and Weatherall, editorial, in N *Engl J Med* 2002); how to maintain the integrity of the scientific record (Smith R.: [editorial]. *BMJ* 2001); and is the university-industrial complex going out of control? (*Nature*, 2001).
18. The 2001 ICMJE revision call for full disclosure of the sponsor's role in research, as well as assurance that investigators are independent of sponsors, are fully accountable for the design and conduct of the trial, have independent access to all trial data and control all editorial and publication decisions. ICMJE has taken an important step in ensuring greater accountability in the research process, its publication and preventing possible malevolent impact on gullible readers. However the findings of a 2002 study suggest that academic institutions routinely participate in clinical research that does not adhere to ICMJE standards of accountability, access to data and control of publication.
19. An issue of major concern is protection of the interests of research subjects because patients agree to become research subjects not only for personal medical benefit but, as an extension, to benefit the rest of the patient population and also advance medical research. These interests are hardly served if research data is doctored or concealed, as can happen to protect industry interests or if industry dictates the terms and conditions of research contracts.
20. The progress of biomedical research depends on ready availability of research subjects. But such ready availability depends on ethical practices by researchers and sponsoring agencies. The clear-cut power to protect research subjects′ interests should be inbuilt in the contract process. Establishment of Best Practice Guidelines for researchers and academic medical centers and Good Publication Practice for sponsoring pharmaceuticals are two important developments worth a close study and replication to assess feasibility across diverse geographical areas.
21. Another area of concern is pharma's focus on the marketability rather than usefulness of products. There is an inevitable slant to produce not necessarily useful but marketable products which ensure profitability of industry and research grants outflow to academia. A disturbing but very relevant finding in this connection is that drugs which can be called “substantial improvements” over available treatments is only, mark the finding, a measly 6%.
22. Industry supports new therapies, not traditional therapy, irrespective of what is effective. Whatever traditional therapy is supported is also most probably because the industry concerned has a product with a big stake there, which has remained a ‘gold standard’ or which that player thinks still has some ‘juice’ left.
23. The larger issue of benefit to society also concerns us here when we realize that industry sponsorship is mainly for potential medications, not for trying to determine whether there may be non-pharmacological interventions that may be equally good, if not better. This is the reason why methods like yoga, psychotherapy, meditation, non-medicated non-mechanised relaxation will not find industry sponsors readily and will never be proved useful apart from anecdotal reporting.
24. In the paradigm shift towards biological psychiatry, the role of industry sponsorship is not overt but probably more pervasive than many have realized or the right thinking may consider good for the health of the branch in the long run. Ask yourself a simple question: Why should industry sponsor psychotherapeutic research? And why should industry *not* sponsor biological research? Which of the two will give rise to drugs? How will the profits pour in? The answer is simple enough.
25. Essentially, there are four types of drugs. First, drugs that work and have minimal side-effects; second, drugs which work but have serious side-effects; third, drugs that do not work but have minimal side-effects; and fourth, drugs which work minimally but have serious side-effects. Pharma's major propelling force can only be producing the first type. They accept the second type only till they can lay their hands on the first and, in any case, never project or accept them as the first. The third type can be occasionally played around with to shore up profits, but never by projecting them as the first type. The fourth type are the laggards, a real threat to credibility and therefore do not deserve any market hype or promotion.
26. What makes pharma adopt even questionable means to make profits? Reasons are mainly three: one, pharma business cannot depend only on genuine discoveries; second, newer drugs with no effect are also with no, or little, side-effects – and that helps; and third, image building needs to balance profit with shored up credibility. Industry players have to strike the right balance between profit making and credibility. In profit making, the marketing champions play their role. In credibility ratings, researchers and paid spokes-persons play their role. All is hunky dory till marketing is based on credibility. When there is nothing available to make for credibility, something is projected as one and marketing carried out, in the calculated hope that profits can accrue, since that must continue endlessly. That is what makes pharma adopt even questionable means to make profits.
27. The process of self-correction set into motion due to greater clout of conscientious researchers, unrelenting expose by medical journalists and supportive editors, patients right activism and law suits against industry will, hopefully, help tilt the balance towards value-based advance, even if belated, and done grudgingly. Major industry players may soften the offensive of such self-correction only by playing the game according to the rules. The earlier the major players understand this, the better it is for all concerned.

